# Health Reporting Characteristics among Journalists in Nepal Utilizing a One Health Framework

**DOI:** 10.3390/ijerph18052784

**Published:** 2021-03-09

**Authors:** Jessica S. Schwind, Stephanie A. Norman, Munshi Khaledur Rahman, Holly L. Richmond, Sameer M. Dixit, Rajesh M. Rajbhandari, Sarah K. Wagner, Dibesh Karmacharya

**Affiliations:** 1 Department of Biostatistics, Epidemiology and Environmental Health Sciences, Jiann-Ping Hsu College of Public Health, Georgia Southern University, Statesboro, GA 30458, USA; hr04848@georgiasouthern.edu (H.L.R.); sw23106@georgiasouthern.edu (S.K.W.); 2 Department of Biostatistics and Epidemiology, Medical College of Georgia, Augusta University, Augusta, GA 30912, USA; whaledoctor@gmail.com; 3 Marine-Med, Bothell, WA 98021, USA; 4 Department of Geology and Geography, College of Science and Mathematics, Georgia Southern University, Statesboro, GA 30458, USA; mkrahman@georgiasouthern.edu; 5 Center for Molecular Dynamics-Nepal, Kathmandu 44600, Nepal; sameer@cmdn.org (S.M.D.); r.rajbhandari@cmdn.org (R.M.R.); dibesh@cmdn.org (D.K.)

**Keywords:** mass media, Nepal, health reporting, One Health, journalists

## Abstract

Journalists play a crucial role in the dissemination of health-related information. In developing countries, such as Nepal, the media are integral in shaping the national agenda and informing the public of important health issues. With an increasing need for a collaborative effort to attain optimal health for people, animals, and the environment, the One Health approach was used to characterize health reporting in Nepal. A comprehensive survey was administered to health journalists regarding their public, animal, and environmental health reporting habits. Seventy-one journalists completed the survey across three study sites. Many journalists indicated a history of reporting across all three sectors but did not routinely focus on health reporting in general. The majority of journalists perceived the quality and overall coverage of health-related topics increased over the last five years. However, few journalists reported receiving specialized training in any health sector. Although the overall quality of health reporting in the Nepali media showed improvements, many journalists acknowledged a lack of understanding of common health topics and a desire to learn more skills related to accurate health reporting. One Health provides a conceptual framework for understanding and promoting health communication through mass media to benefit humans, animals, and ecosystems.

## 1. Introduction

Nepal, a landlocked country in South Asia, is a hotspot area for emerging health threats due to its range of climatic conditions, richness in biodiversity, and high population densities of animals and humans [1,2]. Nepal has experienced significant ecosystem damage caused by natural disasters, such as earthquakes and landslides, and human-made destruction through pollution, overfishing, and a growing human population [3,4]. The presence of complex linkages among human, animal, and environmental health highlights the importance of addressing health threats with all three sectors in mind [5]. In response to these complexities and interactions, the One Health philosophy, a transdisciplinary approach through which organizations promote communication and collaboration among human, animal, and environmental health professionals, was adopted by the greater scientific community to deal with current and future global health challenges [6]. The One Health platform brings together stakeholders in multiple disciplines related to health to solve complex issues that cannot be addressed by any single discipline independently, such as those related to ecosystem function and degradation, disaster preparedness and response, and antimicrobial resistance. One Health also provides an important framework for understanding why some regions are more likely to give rise to spillover events that can lead to emerging infectious diseases in both human and animal populations.

A One Health approach in journalism is important in helping the public understand the linkages described in the One Health philosophy, especially in areas such as Nepal where there is close interaction between humans and animals, both wild and domestic, set against a backdrop of major impacts from climate change [7]. In Nepal, the media transitioned from a government-controlled press to a free press with the National Broadcasting Act of 1993 [8]. As a result, commercialized media took form through the establishment of numerous newspapers and radio stations [9]. Over the past decade, the political atmosphere in Nepal was the primary topic of published news stories, often taking precedence over health reporting [10,11,12]. Health topics rarely made the front page of Nepali newspapers, signifying a perceived lack of importance tied to health reporting across the nation [11,12,13,14]. Despite the far-reaching influence of mass media in developing countries, research examining the impact of health reporting in Nepal has been limited in scope [11,12,15,16]. Past research has suggested positive correlations between mass media campaigns and family planning [17,18] and the utilization of antenatal care services [19], indicating more value should be placed on understanding health communication practices in this evolving media environment.

Mass media serve as important communication channels for relaying health-related information to the public [10,20]. The utilization of print and broadcast media was proven to be an efficient way to reach a breadth of people globally, including those in rural and remote areas [21,22]. The media not only have the capacity to relay current and updated health information to the public but also to effectively influence perceptions, attitudes, behaviors, and policies related to their targeted audience. Media can impact the public perception of a topic’s importance [23], frame how the general public understands a topic [24], and influence health policy associated with a topic [25,26], thus persuading people to adopt healthy behaviors in a variety of global settings [27]. Journalists may even be among the first individuals to bring attention to potential health risks in communities [28].

Understanding journalists’ opinions of the media environment from an inclusive and comprehensive viewpoint, such as the perspective of the One Health framework, is important due to the potential of mass media to set the agenda of individual readers. Should the journalistic point of view become too narrow, so too will their readers. The agenda-setting theory provides a relevant basis to examine the reporting of health-related information. It explains how mass media’s salient portrayal of a topic influences the importance placed on the topic by the general public [29,30]. By making topics more salient, news stories impact the extent to which readers view the topic as noteworthy [31]. Thus, the topical emphasis of a news report, rather than the depiction of the issue itself, was a primary focus of this research. 

While the significance of health communication and communication technologies in public health is well recognized, it is underutilized in veterinary and environmental health sciences [32,33]. A recent study of One Health reporting in Nepal revealed few animal and environmental health media reports were captured by an online, global electronic information system, demonstrating either a low sensitivity for health reports from these sectors or an underreporting of these topics from the Nepali media [34]. Animal and environmental health, however, are directly linked to human health; therefore, the depiction of these in the media is highly relevant to readers and subsequent public knowledge, especially for those in low-resource settings who may not have access to other sources of information. To develop a greater understanding of the Nepali health media coverage from the journalists’ point-of-view, we designed and administered a survey to characterize health reporting habits utilizing a One Health framework. 

## 2. Materials and Methods

**Figure 1:**
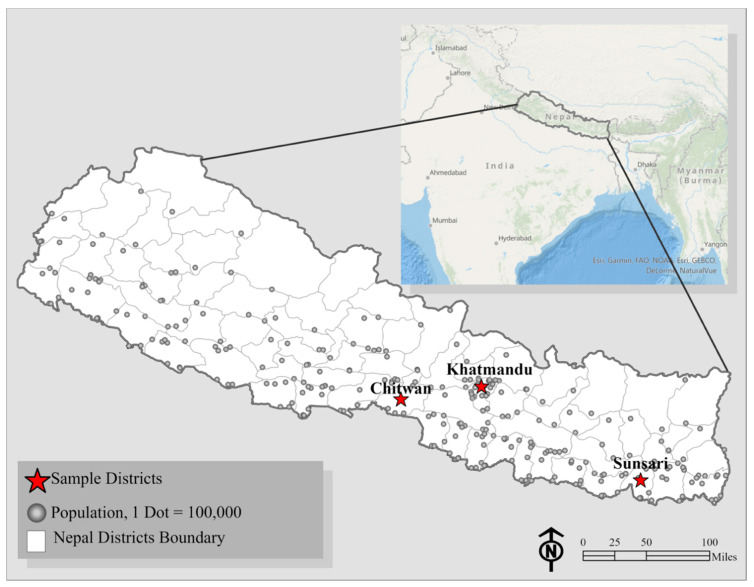
Districts sampled for the journalist survey across Nepal.

A cross-sectional survey tool was developed by our research team and influenced by different journalist surveys, including the Association of Health Care Journalists Member Survey [35] and the Pew Research Center Journalist Survey [36]. The initial survey was first drafted in English, edited as a team, and driven by our research objective to describe the national media environment of health reporting in Nepal with a One Health framework by capturing journalists’ public, animal, and environmental health reporting habits. It was then translated into Nepali through a blind back technique with a focus on ensuring comprehensibility, cultural competency, and One Health applicability. Journalists’ contact information was received from the Ministry of Health and Population in Nepal through a collaboration with the National Health Education, Information and Communication Center. To help obtain a representative sample of health journalists in large population centers with a rich history of environmental and animal health reporting, participants were recruited from three diverse districts (Kathmandu, Chitwan, and Sunsari) across Nepal. The West, Midwest and Far-Western regions were not specifically targeted due to smaller overall populations and a lower number of diverse health journalists in these areas. Sampling locations are shown in Figure 1, along with population densities for reference. Journalists were invited to attend a local information session where the study was introduced, and the survey tool was administered. Potential participants were also encouraged to invite colleagues who frequently reported on health matters. Written informed consent was communicated in English and Nepali, then signed and documented by each participating journalist. Inclusion criteria required participants to be journalists living in Nepal who reported on health events. 

The survey tool consisted of six comprehensive sections, including demographics, general and health reporting habits, focusing on public health, animal health, and environmental health topics. Demographic data collected from the survey tool included age, gender, highest level of education, religion, and average monthly income. General reporting habits explored journalists’ experience, employment, reporting mediums, and preferred reporting language. Health reporting habits included percent effort spent, areas of focus, source contacts, perceived accuracy, perceived barriers, and desired skills. Finally, the public health, animal health, and environmental health sections supplied participants with similar questions that spanned all three health sectors. These questions included the perceived number of articles published per month, primary contacts, prior training experiences, and opinions on the amount and quality of coverage in each health sector.

Data entered into Microsoft^®^ Excel v. 15.30 (Microsoft, Redmond, WA, USA) were analyzed using counts and percentages to describe the study sample in relation to relevant variables. *p*-values were calculated using Chi-square and Fisher’s exact, where applicable, to provide summary statistics for the association between reporting characteristics and a specific health sector. *p*-value < 0.05 was defined as statistically significant. All statistical analyses were conducted using Stata™ v.15.0 (StataCorp, College Station, TX, USA) and performed on de-identified data. This study was approved by the Augusta University Institutional Review Board (#838735) and the Nepal Health Research Council (#281/2015)

## 3. Results

### 3.1. Study Population Characteristics and General Reporting Habits

Only journalists reporting journalistic writing on health topics were included in the study. Initially, 74 journalists completed the survey; however, data from three (4%) journalists were excluded due to an absence of any health reporting experience. A majority of respondents were college-educated (*n* = 43, 60.6%), Hindu (*n* = 67, 90.2%) males (*n* = 57, 80.3%) between the ages of 30 and 39 (*n* = 29, 40.8%), and had an average monthly income of 10,000 to 15,000 Nepalese rupees (NPR; approximately USD 85–128) (*n* = 27, 38.0%) (Table 1). The majority of journalists were directly employed by media companies (*n* = 42, 59.2%) rather than freelance (*n* = 29, 40.8%), and reported across multiple forms of journalistic mediums including a combination of print, broadcast, and web sources (*n* = 35, 49.3%). Furthermore, journalists indicated a considerable preference to reporting in the Nepali language (*n* = 66, 93.0%) over English and Nepali (*n* = 4, 5.6%) or English alone (*n* = 1, 1.4%). Most respondents indicated they have six to fifteen years of journalism experience (*n* = 40, 56.3%) and worked for one to six media sources across their career (*n* = 50, 70.4%) with varying years for their current employer.

### 3.2. Health Reporting Characteristics

There was a wide variation in the percent effort spent on health reporting provided by each journalist (Table 2). Several journalists reported spending 1 to 24% of their total time on health reporting (*n* = 28, 39.4%), followed by 25 to 49% (*n* = 20, 28.2%). Only eleven (15.5%) journalists reported spending a large majority of their time on health reporting. 

Nepali journalists indicated healthcare quality and performance (*n* = 50, 70.4%), consumer/lifestyle health (*n* = 48, 67.6%), and local health issues (*n* = 40, 56.3%) as their primary areas of focus. These responses were followed by the topic areas of health policy (*n* = 28, 39.4%) and health disparities (*n* = 22, 31.0%). Global health issues (*n* = 14, 19.7%), research and science (*n* = 14, 19.7%) and health economics (*n* = 5, 7.0%) were the least reported topics. Journalists reported utilizing personal contacts (*n* = 58, 81.7%) and public or governmental agencies (*n* = 50, 70.4%) for quotes regarding health events. However, a majority of respondents (*n* = 44, 62.0%) reported previously suspecting a colleague of manufacturing quotes regarding a health event. 

Journalists identified several persisting barriers to health reporting in Nepal. Of the total respondents, over two-thirds indicated a lack of knowledge/experience among journalists (*n* = 46, 64.8%) and lack of in-depth coverage (*n* = 45, 63.4%) as the major barriers to health journalism. These responses were followed by a lack of financial resources (*n* = 41, 57.7%) and a lack of personnel (*n* = 39, 54.9%). Additionally, a third of respondents reported the influence of advertisers (*n* = 25, 35.2%) and the influence of political or government officials (*n* = 22, 31.0%) were also a barrier.

Although a lack of knowledge/experience among journalists was the most reported barrier, journalists conveyed a desire for skills to help improve health reporting in Nepal, which included understand and utilize skills in epidemiology terminology (*n* = 52, 73.2%), learn multimedia reporting (*n* = 46, 64.8%), interpret health reports (*n* = 43, 60.6%), and map health conditions and services (*n* = 41, 57.7%). Approximately half of the respondents reported a desire to further understand hospitals or other financial reports (*n* = 35, 49.3%), how to search health information online (*n* = 33, 46.5%), and how to understand opinion polls and surveys (*n* = 32, 45.1%). Lastly, about one-third of the journalists indicated they were interested in obtaining skills to evaluate conflicts of interest (*n* = 28, 39.4%), understand statistics (*n* = 25, 35.2%), and work with Excel or other analytical software (*n* = 24, 33.8%) to improve health reporting in Nepal. 

### 3.3. One Health Reporting Characteristics

From a One Health perspective, a majority of journalists indicated they reported on public health (*n* = 63, 88.7%), animal health (*n* = 59, 83.1%), and environmental health (*n* = 55, 77.5%) topics in the past (Table 3). Furthermore, the majority of journalists who reported in each health sector indicated less than twenty-five health-related articles were published per month on public health (*n* = 37, 58.7%), animal health (*n* = 48, 81.3%), and environmental health (*n* = 39, 0.9%) topics. For journalists reporting in public and environmental health, the greatest number of primary contacts within the health field were researchers with expertise in that particular health sector (*n* = 49, 77.8%; *n* = 49, 89.1%), respectively. Whereas, within the animal health sector, primary contacts were clinical veterinarians (*n* = 39, 66.1%) or other people (*n* = 4, 6.8%) (*p* < 0.0001). 

For each of the three health sectors, the majority of journalists rated the coverage of health events in the Nepali media as poor (*n* = 18, 28.6%; *n* = 28, 47.4%; *n* = 15, 27.3%) to fair (*n* = 34, 53.9%; *n* = 23, 39%; *n* = 30, 54.6%) in public, animal, and environmental health, respectively. Only one respondent in public and environmental health reported health-related media coverage as excellent. In the public and environmental health sectors, most journalists (*n* = 56, 88.9%; *n* = 38, 69.1%, respectively) perceived an increased amount of health-related media coverage at their organizations over the last five years. However, in the animal health sector, a similar number of journalists viewed the amount of health media coverage either stayed the same (*n* = 25, 42.4%) or increased (*n* = 28, 47.4%).

Respondents in the public health sector predominantly reported an increase in the quality of health media coverage within their organization (*n* = 52, 82.5%) over the last five years. However, within the animal and environmental health sectors, the majority of respondents reported either a lesser increase (*n* = 28, 47.4%; *n* = 38, 69.1%) or no change (*n* = 25, 42.4%; *n* = 15, 27.3%) in the quality of the reporting (*p* < 0.0001). Out of the total respondents, half of the journalists (*n* = 32, 50.8%) had not received any training in public health, followed by environmental health (*n* = 29, 52.7%), and animal health (*n* = 44, 74.6 %), respectively (*p* < 0.0001).

## 4. Discussion

The purpose of this study was to provide baseline information on health-related reporting characteristics and practices of journalists in the Nepali media with a distinct focus on human, animal, and environmental health sectors. Seventy-one journalists completed the survey across three study sites, with many indicating a history of reporting across all three sectors, but not a routine focus on health reporting in general. Consistent with what agenda-setting theory would suggest, human health topics were considered more salient than animal health or environmental health topics based on overall media coverage. This study highlighted that knowledge of common health topics and access to financial resources and personnel remain critical concerns for Nepali health media journalists in generating high standards of health stories in all health sectors. These and similar issues have been reported in other regions of the world [37,38]. The majority of journalists perceived the quality and overall coverage of health-related topics has increased over the last five years in Nepal. Empirical studies on health reporting in Nepal or elsewhere are somewhat limited, but available studies suggest that government subsidies for improving health coverage in the media are increasing [12]. Although the overall quality of health reporting in the Nepali media showed improvements, many journalists acknowledged a lack of understanding of common health topics and a desire to learn more skills related to accurate health reporting.

Our study demonstrated that Nepali journalists utilized personal contacts and public or governmental agencies largely for quotes and reported health stories through print or mixed media primarily in the Nepali language. These observations were also seen in other studies where personal connections and use of the local language were defining features of in-country health reporting [39,40]. These findings indicate the importance of the reporter’s social network and the need to promote an interdisciplinary approach to health reporting in general. Communication and collaboration among organizations and professions were identified as critical pillars of One Health [41,42]. As such, journalists should be encouraged to reach across disciplines to bring awareness to the general public of the interconnectedness between humans, animals, and the environment. Establishing a One Health network of professionals, which should include journalists, can lessen barriers associated with health journalism, such as establishing a primary source or contact for health reports. Additionally, respondents in the present study showed that those who reported health-related topics were not specialized health journalists. The respondents identified, instead, as journalists who reported on a variety of news topics, which included health-related issues. Health journalism is unique because consumers rely heavily on this information to inform their own personal- and policy-related health care decisions. This finding agrees with previous reports that indicated trained health journalists in Nepal were sparse and commonly confined to major cities, such as the capital, Kathmandu [12,43,44]. However, it is important to note health disparities are not confined to cities and may disproportionally affect rural and extremely resource-limited communities outside of the capital city [45,46]. In this study, journalists reported on public health topics most frequently, followed by animal health and environmental health, respectively. Our findings were also in line with previous research that revealed a priority on human health event reporting and an underreporting of animal and environmental health events from a global digital epidemiology standpoint in Nepal [34].

This study highlighted differences across health sectors in how journalists perceived the quantity and quality of health reporting over the last five years in Nepal. While most journalists rated the Nepali media coverage of public health, animal health, and environmental health topics as *poor* to *fair*, a large percentage of journalists agreed there was an increase in the amount and quality of media coverage across all three health sectors during the same period. However, this sentiment was not as strong among journalists who reported in the animal health sector. In light of this outlook, past research indicated journalists maintained the responsibility to report on their audience’s needs and issues comprehensively and accurately in Nepal [9] and beyond [20]. Furthermore, Nepali journalists have emphasized the function of journalism and aimed to inform, educate, and advocate the public through the dissemination of their work [9,14,47]. However, challenges and barriers remain, namely, access to resources, time, and capacity constraints, affecting the quality of health reporting in particular [44]. The findings of this study, in general, concur with previous research on journalistic approaches to reporting and supports the notion that numerous factors influence the reporting routines of journalists [48]. There also might be personal influences that play a role in forming decisions by health journalists when reporting on health. For instance, personal interest in a health topic, as well as relationships formed with local public or animal health professionals, may be strong motivators for pursuing a health news story.

In this study, journalists recognized their knowledge and experience as barriers to quality health journalism in Nepal but still reported publishing articles on these topics in the past. Many journalists acknowledged not receiving training in public, environmental, or animal health topic areas, a complaint also reported elsewhere [38]. Research suggests that journalists are often unprepared to cover health-related topics, mainly because they lack adequate expertise on health topics and are not adequately trained in science reporting [49,50]. Yet, journalists reported a desire to further understand and utilize skills associated with epidemiology terminology, multimedia reporting, and the interpretation of health reports. According to Pant (2009), academic journalism programs in Nepal have not kept pace with the fast-changing media environment [9]. Our findings support this research and suggest journalists would benefit from One Health training to further diversify and develop their skillsets, given their tendency to report on a variety of topics. Journalists who author news stories that recognize the interconnectedness of the health of all species may also shape public debate in terms of setting agendas and focusing public interest on more holistic approaches to improving planetary health. In cases where the public has limited knowledge of a particular topic, there is an even greater potential for media influence. For example, an incomplete public understanding of the risks associated with zoonoses could weaken the human-animal bond and negatively impact wildlife conservation, which are both essential components of the One Health movement [51]. Collaboration between journalists and One Health researchers with open lines of communication will certainly aid in achieving this goal. For instance, the One Health European Joint Programme hosts communication and media workshops with a focus on advancing the One Health approach [52]. Inviting and supporting health journalists through these workshops benefit a wide variety of stakeholders including the media, the scientific community, and the populations they serve.

Although this study surveyed journalists in other districts and Kathmandu where prior journalism studies were focused, the limited sample size may not permit generalizability to all journalists who report on health-related topics in Nepal. According to the Federation of Nepali Journalists, there are over 13,000 journalists spread across the country [53], who mainly report on political matters, with a smaller percentage reporting on social issues, of which health topics are included as a subset. Furthermore, it is not clear how many health journalists are in Nepal simply because people do not identify as such, most are not formally trained, and many are informally employed. The survey tool also did not report the number of articles published explicitly by each journalist in each health sector or link this reporting to a particular health outcome. Furthermore, this study relied on self-reporting by journalists and their perceived roles. In such studies, there is always the chance of self-reporting bias by study subjects that may portray more idealized accounts of themselves and their reporting compared to reality [37].

Future research should consider using content analysis to explore how news media frame topics to better understand human, animal, and environmental health discourse in Nepali mass media as a whole. Unlike agenda-setting, framing is concerned with how an issue is depicted in the media and could provide a more robust view of health-related news coverage [54]. Furthermore, the need for enhanced training of health journalists seems clear. Additional efforts should focus on effectively designing and implementing on-the-job training for journalists in Nepal with a focus on One Health. Other practical recommendations to improving health reporting among journalists in Nepal include enhancing the information flow between scientists and health journalists through the sharing of resources (i.e., fact sheets, visual aids, and press releases), raising awareness at professional trade meetings, and developing mentorship programs where health professionals and seasoned health journalists mentor less experienced journalists [44,50]. Finally, health professionals can be strategic in how they work with journalists by strengthening collaborations with the mainstream media to promote the optimal health for people, animals, and the environment.

As globalization and technology advances, media agendas and platforms change. In Nepal, there has been an increase in the use of the internet and social media throughout the country [55]. While our findings illustrated the utilization of traditional media sources, such as print, remain in higher demand than contemporary mediums, journalists’ responsibilities will need to grow to accommodate the challenges caused by this transformation [9,56]. For example, issues over journalistic accountability in online news sources have arisen [57]. These new challenges highlight the need for health journalist training in order to combat misinformation that may be found in non-traditional news sources reporting on health issues. While Nepal made strides toward utilizing contemporary platforms, issues remain with literacy [58] and internet access among audiences, especially those in rural areas [59]. This study may serve as a baseline for shaping future health communication research in Nepal from a One Health perspective. Understanding the theoretical underpinnings of health communication specific to Nepal is an essential component and should be undertaken with a focus on developing models that take into account cultural context and indigenous theory [60,61,62].

As a transdisciplinary field, One Health synthesizes knowledge from across disciplines into one proactive, holistic approach to address planetary health challenges [63]. This approach allows the One Health framework to aid in transdisciplinary thinking and communication about complex systems, which requires multiple voices, unique values, and distinct motivations to propose effective solutions [64]. Though the One Health framework has continued to facilitate the development of coordinated response efforts among its three sectors (human, animal, and environmental health fields) in tackling major health events, communication to the public within a One Health framework has not been as strongly promoted until more recently [65]. By utilizing a One Health approach, journalists can strive to overcome barriers and organizational silos, like those observed in health surveillance systems [66], that can inhibit accurate, holistic health reporting. A well-tailored One Health training program could promote regular media coverage of relevant One Health topics, such as zoonoses, antimicrobial resistance, and food security, as well as address the inexperience and lack of in-depth reporting journalists described in our study.

## 5. Conclusions

With the majority of emerging human infectious diseases originating from zoonotic sources, research at the interface between animals and humans has become progressively more important. Factors such as land-use change and increased globalization have contributed to a resurgence and spread of infectious zoonotic diseases globally. Our study provides powerful insight into the need to promote public, animal, and environmental health articles and the desire to expand their knowledge of One Health among Nepali journalists.

## Figures and Tables

**Table 1:** Study Population Demographics and General Reporting Characteristics.

Variables	*n*	%
Age		
20–29 30–39 40–49 ≥50	24 29 14 4	33.8 40.8 19.7 5.6
Gender		
Male Female	57 14	80.3 19.7
Highest Level of Education		
Secondary education Some college College degree Declined to answer	21 5 43 2	29.6 7.0 60.6 2.8
Religion		
Hindu Buddhist Christian Other	64 4 1 2	90.2 5.6 1.4 2.8
Monthly Income (in Nepali Rupees (NPR))		
<10,000 10,000–15,000 16,000–25,000 26,000–40,000 >40,000	13 27 20 9 2	18.3 38.0 28.2 12.7 2.8
Type of Journalist		
Directly-employed Freelance	42 29	59.2 40.8
Primary Role as a Journalist		
Print journalist Broadcast journalist Web journalist Combination of roles Declined to answer	22 10 2 35 2	31.0 14.1 2.8 49.3 2.8
Primary Reporting Language		
Nepali only Nepali and English English only	66 4 1	93.0 5.6 1.4
Number of Media Sources Worked For		
1–3 4–6 7–10 >10 Declined to answer	24 26 6 6 9	33.8 36.5 8.5 8.5 12.7
Years of Journalism Experience		
≤5 6–10 11–15 16–20 >20	15 19 21 11 5	21.1 26.8 29.6 15.5 7.0
Years with Current Employer		
≤3 4–6 7–10 >10	21 15 18 17	29.6 21.1 25.4 23.9

**Table 2:** Health Reporting Characteristics of the Study Population.

Characteristics	*n*	%
What percent effort is focused on health reporting?		
1–24% 25–49% 50–74% >75% Declined to answer	28 20 8 11 4	39.4 28.2 11.3 15.5 5.6
Areas of focus in health reporting †		
Healthcare quality and performance Consumer/lifestyle health Local health issues Health policy Health disparities Global health issues Research and science Health economics Other	50 48 40 28 22 14 14 5 2	70.4 67.6 56.3 39.4 31.0 19.7 19.7 7.0 2.8
Sources for health reporting topics †		
Personal contacts Public or governmental agencies Other news sources NGOs Online informal sources Unsolicited from public Medical journals Editors	58 50 35 34 33 32 26 19	81.7 70.4 49.3 47.9 46.5 45.1 36.6 26.8
Suspected a colleague manufactured a quote regarding a health event		
Yes No Declined to answer	44 24 3	62.0 33.8 4.2
Barriers most applicable to health journalism in Nepal †		
Lack of knowledge/experience among journalists Lack of in-depth coverage Lack of financial resources Lack of personnel Influence of advertisers Influence of political or government officials Making difficult issues easier to understand Other	46 45 41 39 25 22 14 1	64.8 63.4 57.7 54.9 35.2 31.0 19.7 1.4
Desired skills for health reporting improvement †		
Understand and utilize epidemiology terminology Multimedia reporting Interpret health reports Map health conditions and services Understand hospital or other financial reports How to search for health information online How to understand opinion polls and surveys Evaluate conflicts of interest Understand statistics Work with Excel or other analytical software	52 46 43 41 35 33 32 28 25 24	73.2 64.8 60.6 57.7 49.3 46.5 45.1 39.4 35.2 33.8

† = multiple responses allowed.

**Table 3:** Journalist Reporting Characteristics by Health Sector.

Characteristic	Public Health (63, 88.7%)	Animal Health (59, 83.1%)	Environmental Health (55, 77.5%)
Perceived number of health articles published in this sector per month		
<25 26–50 >50	37 (58.7) 13 (20.6) 13 (20.6)	48 (81.3) 5 (8.5) 6 (10.2)	39 (70.9) 7 (12.7) 9 (16.4)
Primary Contacts *,†		
Government officials NGO representative Researchers with expertise Clinician/veterinarian/other	38 (60.3) 32 (50.8) 49 (77.8) 51 (81.0)	34 (57.6) 20 (33.9) 34 (57.6) 43 (72.9)	37 (67.3) 29 (52.7) 49 (89.1) 1 (1.8)
Rating of Nepali media for health sector coverage		
Poor Fair Good Excellent Declined to answer	18 (28.6) 34 (53.9) 9 (14.3) 1 (1.6) 1 (1.6)	28 (47.4) 23 (39.0) 6 (10.2) 0 (0.0) 2 (3.4)	15 (27.3) 30 (54.6) 8 (14.5) 1 (1.8) 1 (1.8)
Amount of health media coverage at your organization over last 5 years *		
Increased Decreased Stayed the same Declined to answer	56 (88.9) 0 (0.0) 6 (9.5) 1 (1.6)	28 (47.4) 5 (8.5) 25 (42.4) 1 (1.7)	38 (69.1) 0 (0.0) 15 (27.3) 2 (3.6)
Quality of health media coverage at your organization over last 5 years *		
Increased Decreased Stayed the same Declined to answer	52 (82.5) 6 (9.5) 4 (6.3) 1 (1.6)	28 (47.4) 6 (10.2) 21 (35.6) 4 (6.8)	37 (67.3) 2 (3.6) 14 (25.5) 2 (3.6)
Received training in health sector *		
Yes No Declined to answer	28 (44.4) 32 (50.8) 3 (4.8)	6 (10.2) 44 (74.6) 9 (15.2)	23 (41.8) 29 (52.7) 3 (5.5)

* = *p*-value < 0.05; † = multiple responses allowed.

## Data Availability

The datasets analyzed during the current study are available from the corresponding author on reasonable request.

## References

[1] Paudel P.K., Bhattarai B.P., Kindlmann P. (2012). An overview of the biodiversity in Nepal. Himalayan Biodiversity in the Changing World.

[2] Asokan G., Vanitha A. (2016). Disaster response under One Health in the aftermath of Nepal earthquake, 2015. J. Epidemiol. Glob. Heal..

[3] Pandey R., Bardsley D.K. (2015). Social-ecological vulnerability to climate change in the Nepali Himalaya. Appl. Geogr..

[4] Pokhrel D., Bhandari B., Viraraghavan T. (2009). Natural hazards and environmental implications in Nepal. Disaster Prev. Manag. Int. J..

[5] Cunningham A.A., Daszak P., Wood J.L.N. (2017). “One health, emerging infectious diseases and wildlife: Two decades of progress?” Philosophical transactions of the Royal Society of London. Biol. Sci..

[6] Conrad P.A., Meek L.A., Dumit J. (2013). Operationalizing a One Health approach to global health challenges. Comp. Immunol. Microbiol. Infect. Dis..

[7] Otte J., Grace D. (2012). Human health risks from the human-animal interface in Asia. Asian Livest..

[8] Government of Nepal. (1993) The National Broadcasting Act. http://nta.gov.np/wp-content/uploads/2012/06/The-National-Broadcasting-Act1993.pdf.

[9] Pant L.D. (2009). Journalism and media education in Nepal: A critical overview. Bodhi Interdiscip. J..

[10] Gupta A., Sinha A. (2010). Health Coverage in Mass Media: A Content Analysis. J. Commun..

[11] Kumal A.B., Ghimire J., Mishra A., Joshi P., Risal P., Kc R. (2013). Health in Nepalese media. J. Nepal Health Res. Counc..

[12] Tuladhar S., Shresta K.R., Regmi N., Shrestha A., Ban B. (2013). Current Status of Health Reporting in Nepali Press. Bodhi Interdiscip. J..

[13] Adhikari D.N. (2005). Media and democracy in Nepal: A case for public-oriented journalism. Glob. Media J..

[14] Ramaprasad J., Kelly J.D. (2003). Reporting the News from the World’s Rooftop. Gazette.

[15] Thapa S., Mishra V. (2003). Mass media exposure among youth in urban Nepal. Asia Pacific Popul. J..

[16] Van Teijlingen E., Simkhada P., Luce A., Hundley V. (2016). Media, Health & Health Promotion in Nepal. J. Manmohan Mem. Inst. Heal. Sci..

[17] Boulay M., Storey J.D., Sood S. (2002). Indirect Exposure to a Family Planning Mass Media Campaign in Nepal. J. Heal. Commun..

[18] Storey M.B.D. (1999). Impact of the Integrated Radio Communication Project in Nepal, 1994–1997. J. Heal. Commun..

[19] Acharya D., Khanal V., Singh J.K., Adhikari M., Gautam S. (2015). Impact of mass media on the utilization of antenatal care services among women of rural community in Nepal. Bmc Res. Notes.

[20] Schwitzer G., Mudur G., Henry D., Wilson A., Goozner M., Simbra M., Sweet M., Baverstock K.A. (2005). Correction: What Are the Roles and Responsibilities of the Media in Disseminating Health Information?. PLoS Med..

[21] Entwistle V.A., Watt I.S. (1999). Judging journalism: How should the quality of news reporting about clinical interventions be assessed and improved?. Qual. Saf. Heal. Care.

[22] Freimuth V., Cole G., Kirby S.D. (2001). Issues in evaluating mass-media health communication campaigns. WHO Reg. Publ. Eur. Ser..

[23] Tyler T.R., Cook F.L. (1984). The mass media and judgments of risk: Distinguishing impact on personal and societal level judgments. J. Personal. Soc. Psychol..

[24] Entman R.M. (1993). Framing: Toward Clarification of a Fractured Paradigm. J. Commun..

[25] Wallack L. (1994). Media Advocacy: A Strategy for Empowering People and Communities. J. Public Heal. Policy.

[26] Yanovitzky I. (2002). Effects of News Coverage on Policy Attention and Actions. Commun. Res..

[27] Institute of Medicine (2003). Committee on Assuring the Health of the Public in the 21st Century. The Future of the Public’s Health in the 21st Century.

[28] Schwind J.S., Wolking D.J., Brownstein J.S., Mazet J.A.K., Smith W.A. (2014). Evaluation of Local Media Surveillance for Improved Disease Recognition and Monitoring in Global Hotspot Regions. PLoS ONE.

[29] McCombs M.E., Shaw D.L. (1972). The Agenda-Setting Function of Mass Media. Public Opin. Q..

[30] McCombs M. (2005). A Look at Agenda-setting: Past, present and future. J. Stud..

[31] Dearing J.W., Rogers E.M. (1996). Agenda Setting.

[32] Cipolla M., Bonizzi L., Zecconi A. (2015). From “One Health” to “One Communication”: The Contribution of Communication in Veterinary Medicine to Public Health. Veter Sci..

[33] Lapinski M.K., Funk J.A., Moccia L.T. (2015). Recommendations for the role of social science research in One Health. Soc. Sci. Med..

[34] Schwind J.S., Norman S.A., Karmacharya D., Wolking D.J., Dixit S.M., Rajbhandari R.M., Mekaru S.R., Brownstein J.S. (2017). Online surveillance of media health event reporting in Nepal: Digital disease detection from a One Health perspective. BMC Int. Heal. Hum. Rights.

[35] Association of Health Care Journalists Member Survey (n.d.). https://healthjournalism.org.

[36] Center P.R. (2004). Journalist Survey. https://www.journalism.org/2004/03/13/journalist-survey/.

[37] Leask J., Hooker C., King C. (2010). Media coverage of health issues and how to work more effectively with journalists: A qualitative study. Bmc Public Heal..

[38] Voss M. (2002). Checking the Pulse: Midwestern Reporters’ Opinions on Their Ability to Report Health Care News. Am. J. Public Heal..

[39] Sharif A., Medvecky F. (2018). Climate change news reporting in Pakistan: A qualitative analysis of environmental journalists and the barriers they face. J. Sci. Commun..

[40] Veloudaki A., Zota D., Karnaki P., Petralias A., Papasaranti E.S., Spyridis I., Linos A. (2014). Reporting health in Europe: Situation and needs. J. Commun. Heal..

[41] Gibbs E.P.J. (2014). The evolution of One Health: A decade of progress and challenges for the future. Veter. Rec..

[42] Rabinowitz P.M., Kock R., Kachani M., Kunkel R., Thomas J., Gilbert J., Wallace R., Blackmore C., Wong D., Karesh W. (2013). Toward Proof of Concept of a One Health Approach to Disease Prediction and Control. Emerg. Infect. Dis..

[43] Tuladhar S., Shresta K.R., Regmi N., Shrestha A., Ban B. (2013). Assessment of Media Development in Nepal. Based on UNESCO’s Media Development Indicators. UNESCO Kathmandu. https://unesdoc.unesco.org/in/rest/annotationSVC/DownloadWatermarkedAttachment/attach_import_53d178f9-327c-43ea-bc8d-e6eeacd1ac77?_=225486eng.pdf.

[44] Uprety S., Baral S.C., Ghimire R. Capacity building of journalists for urban health reporting in Nepal: An implementation research experience. Proceedings of the Asian Congress for Media and Communication (Acmc).

[45] Andrews J.R., Vaidya K., Bern C., Tamrakar D., Wen S., Madhup S., Shrestha R., Karmacharya B., Amatya B., Koju R. (2017). High Rates of Enteric Fever Diagnosis and Lower Burden of Culture-Confirmed Disease in Peri-urban and Rural Nepal. J. Infect. Dis..

[46] Srinivasan C.S., Zanello G., Shankar B. (2013). Rural-urban disparities in child nutrition in Bangladesh and Nepal. Bmc Public Heal..

[47] Dahal S.P. (2013). Nepalese Journalists’ Democracy Building Roles and News Coverage Practices. Bodhi: Interdiscip. J..

[48] Tanner A.H., Friedman D.B., Zheng Y. (2015). Influences on the Construction of Health News: The Reporting Practices of Local Television News Health Journalists. J. Broadcast. Electron. Media.

[49] Massimo C. (2012). Communication and miscommunication of public-health risks: Towards good practice for medical journalism. J. Mass Commun. J..

[50] Lowrey W., Evans W., Gower K.K., Robinson J.A., Ginter P.M., McCormick L.C., Abdolrasulnia M. (2007). Effective media communication of disasters: Pressing problems and recommendations. Bmc Public Heal..

[51] Decker D.J., Evensen D.T., Siemer W.F., Leong K.M., Riley S.J., Wild M.A., Castle K.T., Higgins C.L. (2010). Understanding Risk Perceptions to Enhance Communication about Human-Wildlife Interactions and the Impacts of Zoonotic Disease. Ilar J..

[52] One Health European Joint Programme. Communication and Media Work-Shop. https://onehealthejp.eu/communication-and-media-workshop/.

[53] Federation of Nepali Journalists (n.d.). http://fnjnepal.org/en/page/about-fnj.

[54] Weaver D.H. (2007). Thoughts on agenda setting, framing, and priming. J. Commun..

[55] Kshetri I.D. (2008). Online News Portals in Nepal: An Overview. Bodhi Interdiscip. J..

[56] Paek H.-J., Lee A.L., Jeong S.-H., Wang J., Dutta M.J. (2010). The Emerging Landscape of Health Communication in Asia: Theoretical Contributions, Methodological Questions, and Applied Collaborations. Heal. Commun..

[57] Acharya B.B. (2019). Accountability in Online News Media: A Case Study of Nepal. Athens J. Mass Media Commun..

[58] Robinson-Pant A. (2010). Changing discourses: Literacy and development in Nepal. Int. J. Educ. Dev..

[59] Regmi N. (2017). Expectations versus Reality: A Case of Internet in Nepal. Electron. J. Inf. Syst. Dev. Ctries..

[60] Haslett B.B. (2017). East and west communication: A framework for dialogue. China Media Res..

[61] Lwin M.O., Salmon C.T. (2015). A retrospective overview of health communication studies in Asia from 2000 to 2013. Asian J. Commun..

[62] Wang G., Kuo E.C. (2010). The Asian communication debate: Culture-specificity, culture-generality, and beyond. Asian J. Commun..

[63] Kelly T.R., Karesh W.B., Johnson C.K., Gilardi K.V., Anthony S.J., Goldstein T., Olson S.H., Machalaba C., Mazet J.A. (2017). One Health proof of concept: Bringing a transdisciplinary approach to surveillance for zoonotic viruses at the human-wild animal interface. Prev. Veter. Med..

[64] Deem S.L., Lane-deGraaf K.E., Rayhel E.A. (2019). Introduction to One Health: An Interdisciplinary Approach to Planetary Health.

[65] Jemison M. (2017). Communication considerations for One Health: The influence of media framing on representations of a human-bat disease conflict in the Australian print media. Aust. Zool..

[66] Stärk K.D., Kuribreña M.A., Dauphin G., Vokaty S., Ward M.P., Wieland B., Lindberg A. (2015). One Health surveillance–More than a buzz word?. Prev. Veter. Med..

